# Early Onset Bipolar Disorder in a 5.5 Years- Old Child

**Published:** 2011

**Authors:** Mina Zarei, Reza Bidaki, Mitra Hakim-Shooshtari

**Affiliations:** 1 Tehran University of Medical Sciences,; 2 Rafsanjan University of Medical Sciences

**Keywords:** Bipolar disorder, Childhood, Familial History

## Abstract

Bipolar disorder is a mental disease that can be presented as irritable mood with affective storms, mixed symptoms of depression and mania, rapid cycles, emotional labiality and irritability during all episodes. A confirmed positive familial history of the disease is the single most robust risk factor for developing the illness. This report presents 5.5 years-old girl with the symptoms of bipolar disorder and with the purpose to draw attention to the diversity of possible symptoms of mood disorders in childhood.

## Introduction

Over the past decade, Bipolar mood disorder (BD) gets recognized as a pathology that could be presented in the younger population (1). BD is a controversial diagnosis, and its exact clinical characteristics are subject to significant debate (2,3). The incidence of BD is estimated between 0.2% and 0.4% and it demonstrates the pediatric BD as a significant public health problem. In children and adolescents, symptoms can have different meanings based on the developmental level of the child (4).

Irritability and emotional liability have been described as the most common symptoms, while elated mood and grandiosity have been described as the most cardinal symptoms in pediatric BD (2). In these patients BD often overlaps or occurs in combination with other disorders such as Attention-deficit/hyperactivity disorder (ADHD) in 80-90% of children, depression and anxiety (5). A confirmed positive familial history is the single most robust risk factor for developing the illness given a high heritability (6). Pediatric BD may become a more serious problem in the future, because of the age of its onset which may be getting younger in more recent birth cohorts (7). The present report describes a case of bipolar disorder with an onset in childhood.

## Case Report

In May 2009, a 5.5 years old girl was refereed to the child psychiatric clinic for further assessments. She was born in Afghanistan and until the time of gathering data she was living in Iran. She had two siblings and she was the youngest one. They had no physical and/or mental abnormalities. Their parents were consanguineous and they had no abnormalities either. The patient’s uncle was diagnosed with BD several years ago. The socio economic level of the family was low. The patient didn't have any past psychiatric or medical problems. Her symptoms started about 6 months ago by some rapid cycles of mood swings and she showed marked mood liability, distractibility and rages and explosive temper tantrums (lasting up to several hours). At each episode she was starting to laugh most of the time even whole night for about two weeks. She didn't show talkativeness and impairment in perception or thought. After passing this phase, she was crying with the same pattern for another two weeks. Her behavior was clingy and her appetite has decreased during the depressive phases. Meanwhile she has started to scare while sleeping and also using the bathroom or toilet, this situation got severe since 2 months ago. Then after a while she also scared from other people and children; she wanted to be hugged and being protected by her parents all the times. Since then she always repeated the sentence “I want to be clean”, but she showed no compulsive symptoms. Based on these symptoms she had a separation anxiety problem but the parents didn't seek any help for her problem. Her attention span had decreased. She was also very aggressive and had marked irritability and she bit her nails. She had distractibility, hyperactivity and labile emotion and also restlessness and fidgetiness. Her sleep was decreased as well. Her social interaction was poor. She had no oppositional behavior, racing thoughts or grandiosity. She also had no difficulty getting up in the morning, bed wetting or night terrors. There were no signs or symptoms of compulsive behavior, tics, paranoia, hallucinations and delusions in her. The parents didn't mention any cruelty to animals in her. She didn't have any suicidal ideation. The child's developmental history was normal but her emotional development was impaired. About 6 months ago she had visited by a general psychiatrist and risperidon was prescribed for her but she refused to get it. There is no report about the diagnosis on that time. At our clinic, a child psychiatrist and a general psychiatrist visited her. She had a typical full-blown BD that rarely is seen in the children. The diagnosis was based on DSM-IV-TR criteria for BD. There were no comorbidities. Based on our diagnosis, we strongly suggested treating her at in-patient psychiatric setting, but her family refused to admit her at the hospital.

During our observation she had severe mood labiality, behavioral disturbances and decreased need to sleep. She didn't accept to eat tablet, therefore the only mood stabilizer that is accessible in syrup form (valprovate 200mg/day) was prescribed. Her biochemistry and thyroid function tests were normal. No impairment was reported in EEG and brain imaging. In June 2009, we followed her up by speaking to her mother via telephone. The patient had a very poor compliance and didn't take her medication so she didn't have any changes in her behavioral problems but her mood labiality got milder without any medication. In fact it is accounted as a form of child neglect which parents ignore the right of child to receive the treatment.

## Discussion

Accompanying the striking increase in the frequency with which pediatric BD is being diagnosed (4), there has been significant controversy and confusion surrounding the definition, assessment, and treatment of this condition. The interesting aspect of our case is that she has experienced several distinct episodes with different moods. Changing vegetative symptoms is the other interesting point that often does not happen at childhood (8) which has happened in our patient. Consanguineous parents and the history of BD in her uncle are the important factors that probably led to the low age of BD onset in this patient. Pauls et al state that when BD starts at childhood, the patient is more likely to have a positive familial history of the disorder (9).

Distinguishing between BD and other conditions of childhood is complicated by overlapping symptoms and by the confounding influences of development itself. At the center of the debate regarding symptom overlap has been a focus on differentiating between symptoms of ADHD and BD (10).

Few guidelines have been formulated for pharmacological treatment of BD, particularly in children and adolescents. Controlled studies with small samples have provided data supporting the efficacy of valproate, lithium, and carbamazepine (11). In this case we used valproate to treat the patients and we observed an acceptable response to this medication despite of the poor compliance of the patient.

Detailed information regarding history and longitudinal course of symptoms is necessary to diagnose a mood disorder. In many instances, diagnostic clarity may only come with extended longitudinal follow-up. The early diagnosis of this disorder in children is important to be noticed by general practitioners to refer these patients to psychiatrist to treat in the best way without wasting any time.

## Authors' contributions

MZ conceived and designed the evaluation and drafted the manuscript. RB participated in designing the evaluation. MH-S re-evaluated the clinical data and revised the manuscript. All authors read and approved the final manuscript

## Notes

**Declaration of interest**: None.
